# Autophagy protein Atg7 is essential for maintaining malaria parasite cellular homeostasis and organelle biogenesis

**DOI:** 10.1128/mbio.02735-24

**Published:** 2024-12-23

**Authors:** Akancha Mishra, Suryansh Rajput, Pratik Narain Srivastava, H. Shabeer Ali, Satish Mishra

**Affiliations:** 1 Division of Molecular Microbiology and Immunology, CSIR-Central Drug Research Institute, Lucknow, India; 2 Academy of Scientific and Innovative Research (AcSIR) 550336 , Ghaziabad, India; The George Washington University Milken Institute of Public Health, Washington, DC, USA

**Keywords:** Atg7, Atg8, apicoplast, ER, autophagy, *Plasmodium*, malaria, sporozoites, liver stage

## Abstract

*Plasmodium* parasites have a complex life cycle that transitions between mosquito and mammalian hosts, and undergo continuous cellular remodeling to adapt to various drastic environments. Following hepatocyte invasion, the parasite discards superfluous organelles for intracellular replication, and the remnant organelles undergo extensive branching and mature into hepatic merozoites. Autophagy is a ubiquitous eukaryotic process that permits the recycling of intracellular components. Here, we show that the *Plasmodium berghei* autophagy-related E1-like enzyme Atg7 is expressed in the blood, sporozoites, and liver stages, localized to the parasite cytosol, and is essential for the localization of Atg8 on the membrane and the development of parasite blood and liver forms. We found that depleting Atg7 abolishes Atg8 lipidation, exocytosis of micronemes, organelle biogenesis, and the formation of merozoites during liver-stage development. Overall, this study establishes the essential functions of Atg7 in *Plasmodium* blood and liver stages, and highlights its role in maintaining the parasite’s cellular homeostasis and organelle biogenesis.

**IMPORTANCE:**

The malaria life cycle involves two hosts, mosquitoes and vertebrates. *Plasmodium* parasites undergo complex intracellular and extracellular stages during this transition. Here, we report that an autophagy-related E1-like enzyme Atg7 is required to conjugate Atg8 on the apicoplast membrane. Atg7 depletion in *Plasmodium berghei* resulted in the loss of Atg8 lipidation and multiple defects like clearance of micronemes, organelle biogenesis, and maturation of hepatic schizonts during liver-stage development. The essentiality of *Plasmodium* Atg7 in blood and liver stages suggests it is a prospective target for developing autophagy-specific inhibitors. These results highlight the importance of autophagy in malaria parasite development.

## INTRODUCTION

The sporozoites invade hepatocytes and undergo extreme metamorphosis by completely dismantling the inner membrane complex (IMC), eliminating the invasive secretory organelles, and achieving an exoerythrocytic form (EEF) (1, 2). The EEF divides its nucleus several times, and the remnant organelles undergo extensive branching and mature into hepatic merozoites (3). These merozoites are released in the bloodstream as merosomes, where upon rupture, each merozoite invades and divides in RBCs (3, 4). Throughout this development process, the parasite eliminates unnecessary superfluous organelles (1). Understanding this process may provide insights into the parasite’s smooth transitions and development in different stages.

Autophagy (macroautophagy) is one such catabolic process widely conserved among eukaryotes and is induced in response to numerous cellular stresses, such as starvation, hypoxia, cellular differentiation, protein metabolism, invading pathogens, and aging (5–9). This involves isolating cytoplasmic components in a double membranous vesicle known as an autophagosome that later fuses and delivers its content to lysosomes for degradation (10). Lipids, amino acids, and other macromolecules generated after lysosome degradation are later recycled to maintain cellular homeostasis (11). Approximately 35 autophagy-related genes that have been characterized thus far in yeast are required for maintaining autophagy at various steps (12). Bioinformatic studies found partial conservation of ATG genes in apicomplexan parasites (13). Parasites lack the genes required for upstream sensing and signaling for the induction of autophagy but have genes needed for membrane expansion and completion. The redundant set of Atgs has also diversified their role more toward sustenance and aiding in extensive replication of the parasites in the host (14).

Atg8, a marker of active autophagy in yeast and mammals, is a small ubiquitin-like molecule, and its conjugation with phosphatidyl ethanolamine (PE) on the autophagosome membrane is essential for cargo selection, membrane tethering, hemifusion, expansion, and closure of autophagosomes (15–18). In apicomplexan parasites, Atg8 is conserved along with the intermediate genes required for its conjugation with PE and is specifically localized on the apicoplast membrane (19, 20). The discrete localization of Atg8 on the apicoplast membrane throughout the parasite life cycle implies it has a distinct role from its role in autophagy (21). In a recent study, the loss of Atg8 function led to the complete loss of apicoplast and arrested parasite growth in *Plasmodium falciparum* (21). We previously showed that hampering the deconjugation of Atg8 on the apicoplast membrane affected its biogenesis and parasite maturation in the liver (22). Similar defects in apicoplast biogenesis were observed in *Toxoplasma gondii* Atg8 knockdown, leading to a parasite replication block (23). Targeting Atg7 (E1 activating enzyme), which is required for Atg8 conjugation, attenuated parasite growth in *P. falciparum* blood stages (19, 24, 25). Similar phenotypes of growth attenuation were observed in the *TgAtg4* and *TgAtg3* knockdowns, depicting the essentiality of the pathway in the parasite survival (26, 27).

Previous reports suggested a role for Atg8 in *P. falciparum* blood-stage development (14, 19); however, the importance of the Atg8 conjugation system in liver-stage development remains largely unknown (24, 25). This study demonstrates the role of the autophagy-related protein Atg7 in the *Plasmodium berghei* life cycle, focusing on its role in liver-stage development. The Atg7 gene was found to be indispensable in *P. berghei* blood stages. By utilizing Flp/FRT-based conditional mutagenesis, we elegantly demonstrate its role in Atg8 conjugation, clearance of micronemes, organelle biogenesis, and expansion of parasites during liver-stage development.

## RESULTS

### Atg7 is expressed during asexual blood, sporozoites, and liver stages and localized to the parasite cytosol

**Fig 1:**
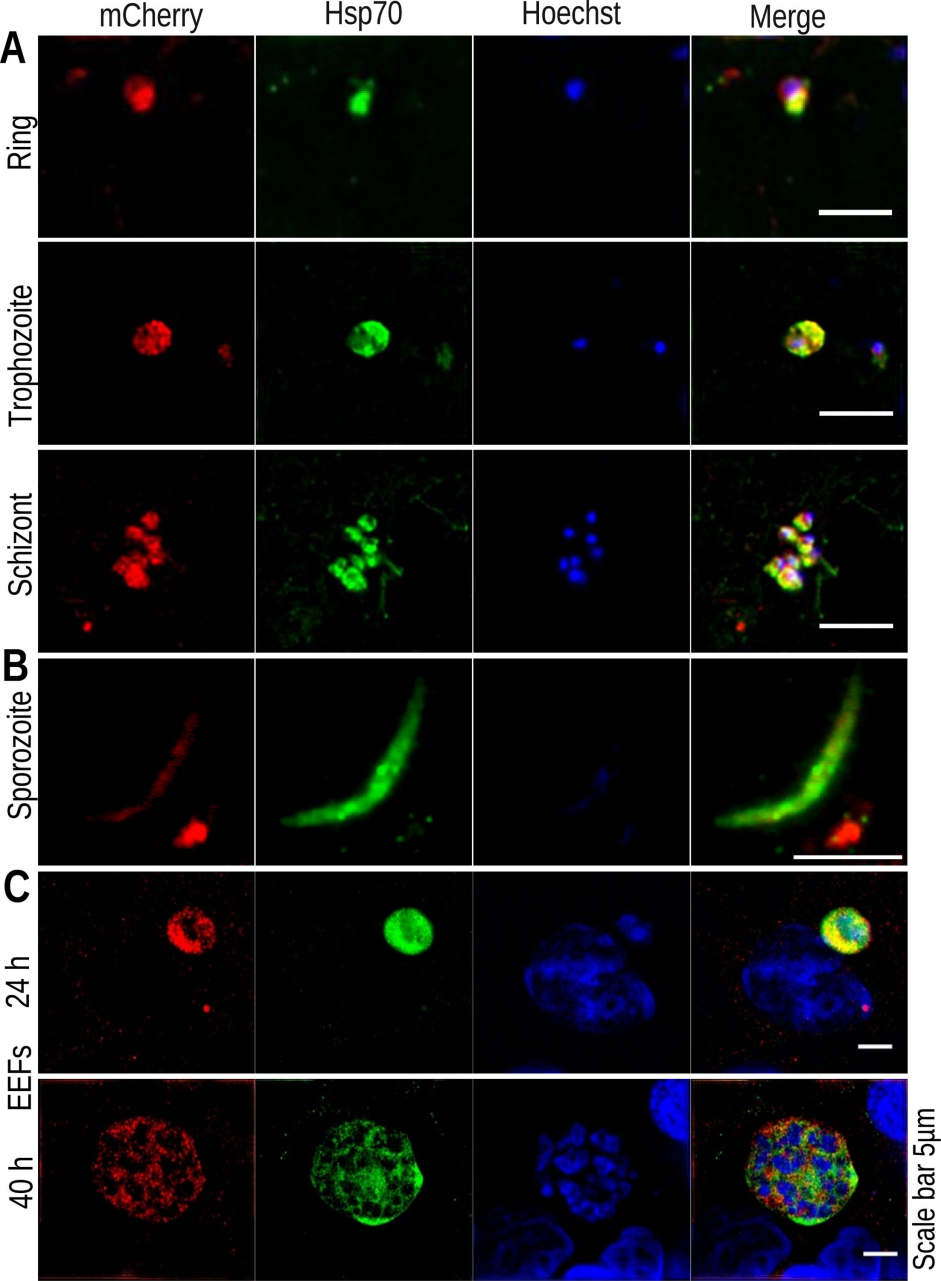
Atg7 expression and localization. (**A**) Atg7-3×HA-mCherry-infected blood-stage slides were prepared, fixed, and immunostained with anti-Hsp70 and anti-mCherry antibodies. Merged images show the colocalization of Atg7 with Hsp70 in the ring, trophozoite, and schizonts, indicating the cytosolic expression and localization of Atg7 in blood stages with PCC values of 0.672, 0.929, and 0.929, respectively. (**B**) Sporozoites were fixed and immunostained with anti-Hsp70 and anti-mCherry antibodies. Images show the colocalization of Atg7 and Hsp70 with a PCC value of 0.672. (**C**) HepG2 cells infected with Atg7-3×HA-mCherry sporozoites were fixed at 24 and 40 hpi, and stained with anti-Hsp70 and anti-mCherry antibodies. Similar to blood stages, Atg7 was expressed in the EEF cytosol. The Atg7 and Hsp70 signals colocalized at both time points (24 and 40 hpi) with PCC values of 0.775 and 0.444, respectively. The nuclei were stained with Hoechst 33342.

We began our study by evaluating the spatiotemporal localization of Atg7 at various stages of parasite development. We generated a transgenic parasite line that endogenously expresses the *Atg7*-3×HA-mCherry fusion protein (see Fig. S1A and B at https://doi.org/10.6084/m9.figshare.27641523.v2). The *Atg7*-3×HA-mCherry parasite line completed the life cycle normally, indicating that Atg7 was unaffected by tagging (see Fig. S1C and D at https://doi.org/10.6084/m9.figshare.27641523.v2). For expression analysis, blood-stage parasites were immunostained with anti-mCherry and anti-Hsp70 antibodies. mCherry signals showed the cytosolic localization of Atg7 in the schizont, ring, and trophozoite stages, with Pearson correlation coefficient (PCC) values of 0.672, 0.929, and 0.929, respectively (Fig. 1A). Live mCherry signals were not observed during mosquito stages. Next, we immunostained *Atg7*-3×HA-mCherry sporozoites with anti-mCherry and anti-Hsp70 antibodies, which revealed cytosolic expression of Atg7 (Fig. 1B). For liver-stage expression analysis, HepG2 cells were infected with *Atg7*-3×HA-mCherry sporozoites, and EEFs harvested at different time points were immunostained with anti-mCherry and anti-Hsp70 antibodies. Immunofluorescence assay (IFA) revealed cytosolic localization with PCC values of 0.775 and 0.444 at 24 and 40 hours postinfection (hpi), respectively (Fig. 1C). The mCherry signals were not detected at 55 hpi, revealing the early to mid-liver-stage expression of Atg7 protein.

### Atg7 is essential for the development of *P. berghei* blood and liver stages

**Fig 2:**
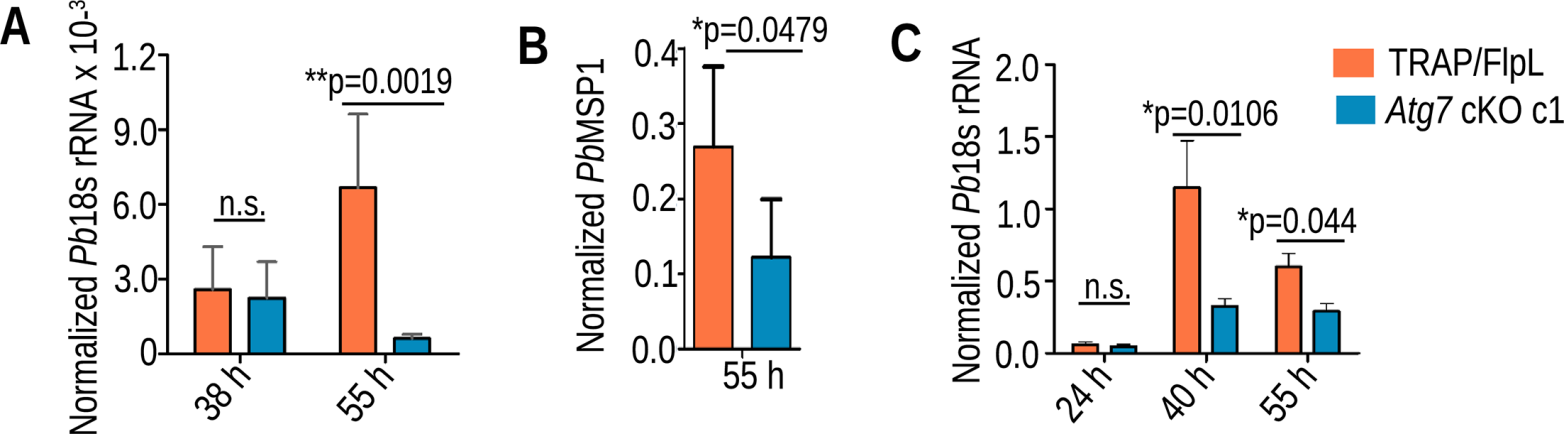
Infectivity of *Atg7* cKO sporozoites in C57BL/6 mice. (**A**) Real-time analysis of parasite burden in the liver showing the comparison of normalized *Pb*18s rRNA transcript numbers in TRAP/FlpL- and *Atg7* cKO-infected mice at 38 and 55 hpi. Data representative of three independent experiments are presented as the mean ± SD, *n* = 5 (no significant difference, 38 h [*P* = 0.9933], significantly different at 55 h [*P* = 0.0019], Student’s *t*-test). (**B**) Quantification of *PbMSP1* transcript numbers in TRAP Flp/L- and *Atg7* cKO-infected mouse livers harvested at 55 hpi. Data representative of three independent experiments are presented as the mean ± SD, *n* = 5 (statistically significant [*P* = 0.0479], Student’s *t*-test). (**C**) Quantification of parasite burden in infected HepG2 cells harvested at 24, 40, and 55 hpi. Data are presented as the mean ± SEM, *n* = 3 biological replicates (no significant difference at 24 h [*P* = 0.6194], significant difference at 40 h [*P* = 0.0106] and 55 h [*P* = 0.044], Student’s *t*-test).

**Fig 3:**
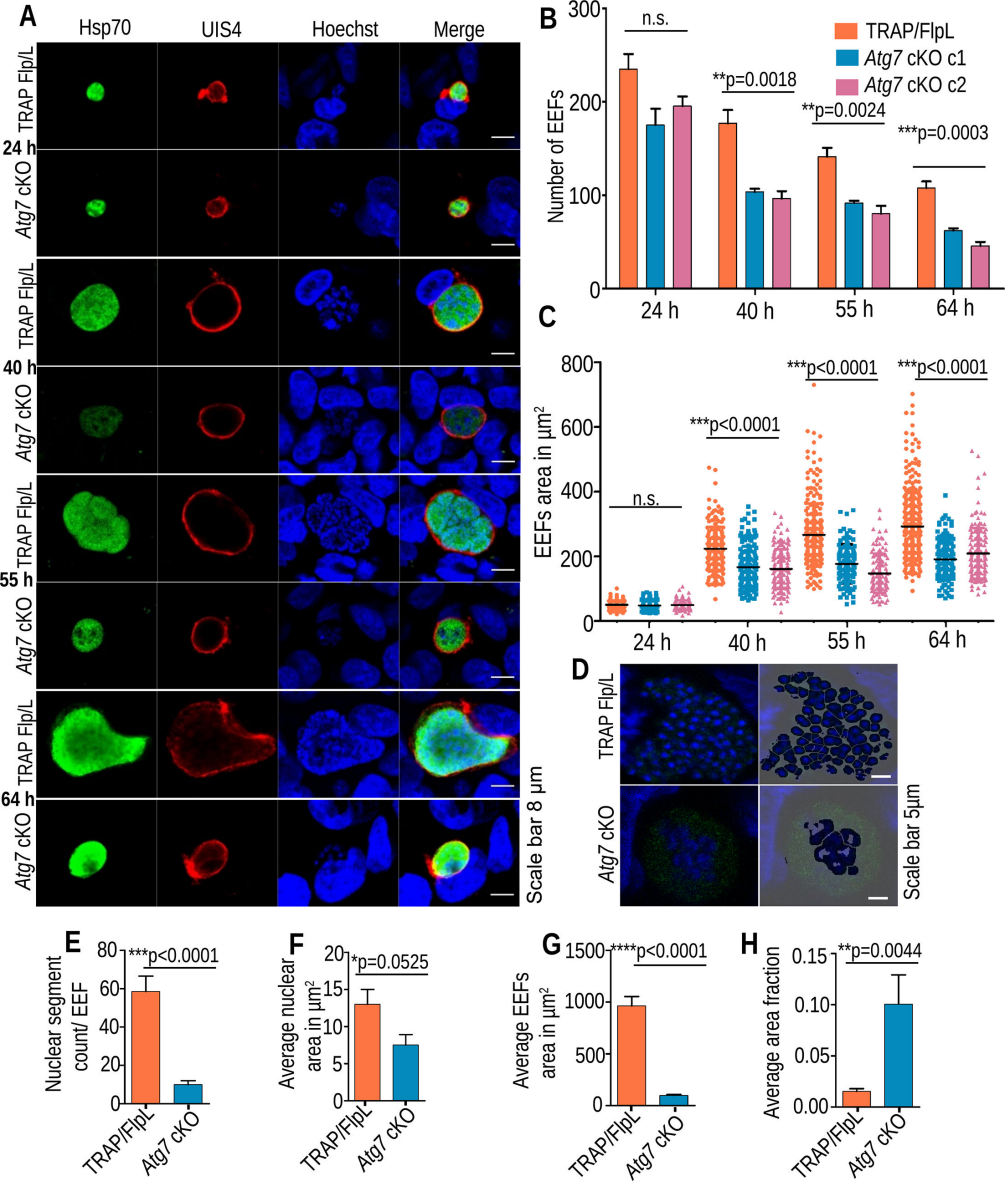
Atg7 is essential for late liver-stage development. (**A**) HepG2 cells infected with *Atg7* cKO or TRAP Flp/L sporozoites were fixed at 24, 40, 55, and 64 hpi and analyzed by IFA. Parasites were stained with anti-UIS4 and anti-Hsp70 antibodies. Nuclei were stained with Hoechst 33342. (**B**) The number of EEFs was quantified and is presented as the mean ± SEM. *n* = 3 biological replicates (no significant difference at 24 h [*P* = 0.0760], significant difference at 40 h [*P* = 0.0018], 55 h [*P* = 0.0024], and 64 h [*P* = 0.0003], one-way ANOVA). (**C**) Measurement of EEF area at 24, 40, 55, and 64 hpi. Data were pooled from three independent experiments. *n* = 150 to 200 EEFs per parasite strain (no significant difference at 24 h, *P* = 0.3055; significant difference at 40, 55, and 64 h [*P* < 0.0001], one-way ANOVA). (**D**) *Atg7* cKO parasites have compromised nuclear segmentation. TRAP/FlpL and *Atg7* cKO EEFs fixed at 64 h were identified by the Hsp70 antibody, and the nucleus was stained with Hoechst 33342. (**E**) Nuclear segments count in TRAP/FlpL and *Atg7* cKO EEFs. (**F**) Average nuclear area of TRAP/FlpL and *Atg7* cKO EEFs. (**G**) Average EEF area of TRAP/FlpL and *Atg7* cKO parasites. (**H**) Average nuclear/EEF area fraction in TRAP/FlpL and *Atg7* cKO EEFs. Data are presented as the mean ± SEM. *n* = 16, from three biological replicates. The Student’s *t*-test was used to compare the difference between TRAP/FlpL and *Atg7* cKO.

To decipher Atg7 function in the *Plasmodium* life cycle, we attempted to disrupt Atg7 three times by double crossover homologous recombination directly but failed to obtain knockout (KO) parasites in the blood-stage (see Fig. S2A at https://doi.org/10.6084/m9.figshare.27641523.v2). Next, we employed the yeast Flp/FRT-based conditional mutagenesis system to silence Atg7 function in sporozoites (28). We engineered two FRT sites so that after excision in mosquito stages, a portion of the gene was excised (see Fig. S2B through E at https://doi.org/10.6084/m9.figshare.27641523.v2). The construct was also transfected into *P. berghei* wild-type (WT) parasites to ensure that the excision of the flirted Atg7 locus was specific to the TRAP/FlpL line (see Fig. S2F at https://doi.org/10.6084/m9.figshare.27641523.v2). These genetic manipulations did not affect parasite development in the blood and mosquito stages (see Fig. S3A through E at https://doi.org/10.6084/m9.figshare.27641523.v2). Atg7 cKO is identical to the control parasite before the gene excision, which occurs in sporozoites. Hence, we recorded late (mature) oocysts still surviving in the midgut on day 14 post-blood meal and also monitored the sporogony. To check the excision efficiency of the *Atg7* gene in sporozoites, genotyping was performed, which revealed successful excision of the flirted *Atg7* locus transfected in the TRAP/FlpL line but not in the *P. berghei* WT parasites, which do not express FlpL (see Fig. S3F at https://doi.org/10.6084/m9.figshare.27641523.v2). Next, we checked the transcripts of Atg7 in TRAP/FlpL and *Atg7* cKO midgut and salivary gland sporozoites. The transcripts were decreased in midgut sporozoites and could not be detected in the salivary gland sporozoites (see Fig. S3G at https://doi.org/10.6084/m9.figshare.27641523.v2). These results demonstrate that *Atg7* cKO parasites failed to express Atg7 transcripts after the excision of the locus. Possibly, Atg7 is not required for the progression of sporozoites from the midgut to the salivary gland. However, we cannot rule out the possibility of Atg7’s role during gametogenesis to sporogony in mosquito midgut. A conditional mutagenesis system that silences the gene function in gametocyte stages may reveal the role of Atg7 during mosquito stages. Next, sporozoites were intravenously injected into C57BL/6 mice, and the prepatent period was observed. We found that all the mice inoculated with TRAP/FlpL or *Atg7* cKO/WT sporozoites became patent on day 3 postinfection (p.i.), whereas parasites lacking Atg7 (*Atg7* cKO) failed to initiate blood-stage infection (Table 1). We observed blood-stage infection in 46% of infected mice, and genotyping revealed a non-excised *Atg7* locus (see Fig. S3F and H at https://doi.org/10.6084/m9.figshare.27641523.v2). This indicates that few parasites that escape the excision of their flirted locus in the mosquito stages were able to complete liver-stage development and initiate blood-stage infection. To identify the stage-specific roles of Atg7, C57BL/6 mice were injected intravenously (i.v.) with sporozoites, and parasite burden in the liver was quantified by real-time PCR analysis of *Pb*18S rRNA transcripts. We found comparable parasite burden in *Atg7* cKO and TRAP/FlpL parasites until 38 hpi, which was decreased in late time point samples harvested at 55 hpi (Fig. 2A). As the parasites undergo schizogony, they start expressing merozoite surface protein 1 (MSP1), whose product is important for merozoite formation. We found a significant decrease in MSP1 transcripts in *Atg7* cKO parasites at 55 hpi (Fig. 2B). During exoerythrocytic schizogony, parasites undergo multiple rounds of nuclear division, increase in size, and mature into infectious merozoites that fill the sac called the merosome. To visualize this development pattern in *Atg7* cKO parasites, HepG2 cultures were infected with sporozoites and harvested at different time points. *Atg7* cKO sporozoites invaded hepatocytes normally (see Fig. S4A and B at https://doi.org/10.6084/m9.figshare.27641523.v2). The parasites in the infected HepG2 cells were quantified using qRT‒PCR, which was consistent with the *in vivo* liver infectivity (Fig. 2C). Immunofluorescence analysis of EEFs with anti-Hsp70 and anti-UIS4 antibodies followed by quantification revealed normal development until 24 hpi. However, EEFs harvested at 40, 55, and 64 hpi showed arrested growth and reduced size and number (Fig. 3A through C). On quantifying the Hoechst-stained DNA centers, we also observed a strong defect in nuclear fragmentation in Atg7 cKO parasites at late time points and a significantly increased nuclear/EEF area fraction (Fig. 3D through H).

**TABLE 1:** Infectivity of *Atg7* cKO sporozoites in C57BL/6 mice^*a*^

Experiment	Parasites	Number of sporozoites injected	Mice positive/mice injected	Pre-patent period (days)
1	TRAP/FlpL	5,000	3/3	3
	*Atg7* cKO SC1	5,000	4/5	5.75
2	TRAP/FlpL	5,000	3/3	3
	*Atg7* cKO SC1	5,000	1/5	5
3	TRAP/FlpL	5,000	3/3	3
	*Atg7* cKO SC1	5,000	1/5	6
4	TRAP/FlpL	5,000	3/3	3
	*Atg7* cKO/WT	5,000	5/5	3
	*Atg7* cKO SC2	5,000	4/5	5.5
5	TRAP/FlpL	5,000	3/3	3
	*Atg7* cKO/WT	5,000	5/5	3
	*Atg7* cKO SC2	5,000	1/4	5

^*a*^ Mice were i.v. inoculated with *Atg7* cKO, TRAP/FlpL, or Atg7 cKO/WT sporozoites. SC1 and SC2 represent single clones 1 and 2.

### *Atg7* cKO parasites failed to mature into hepatic merozoites due to impaired organelle biogenesis

**Fig 4:**
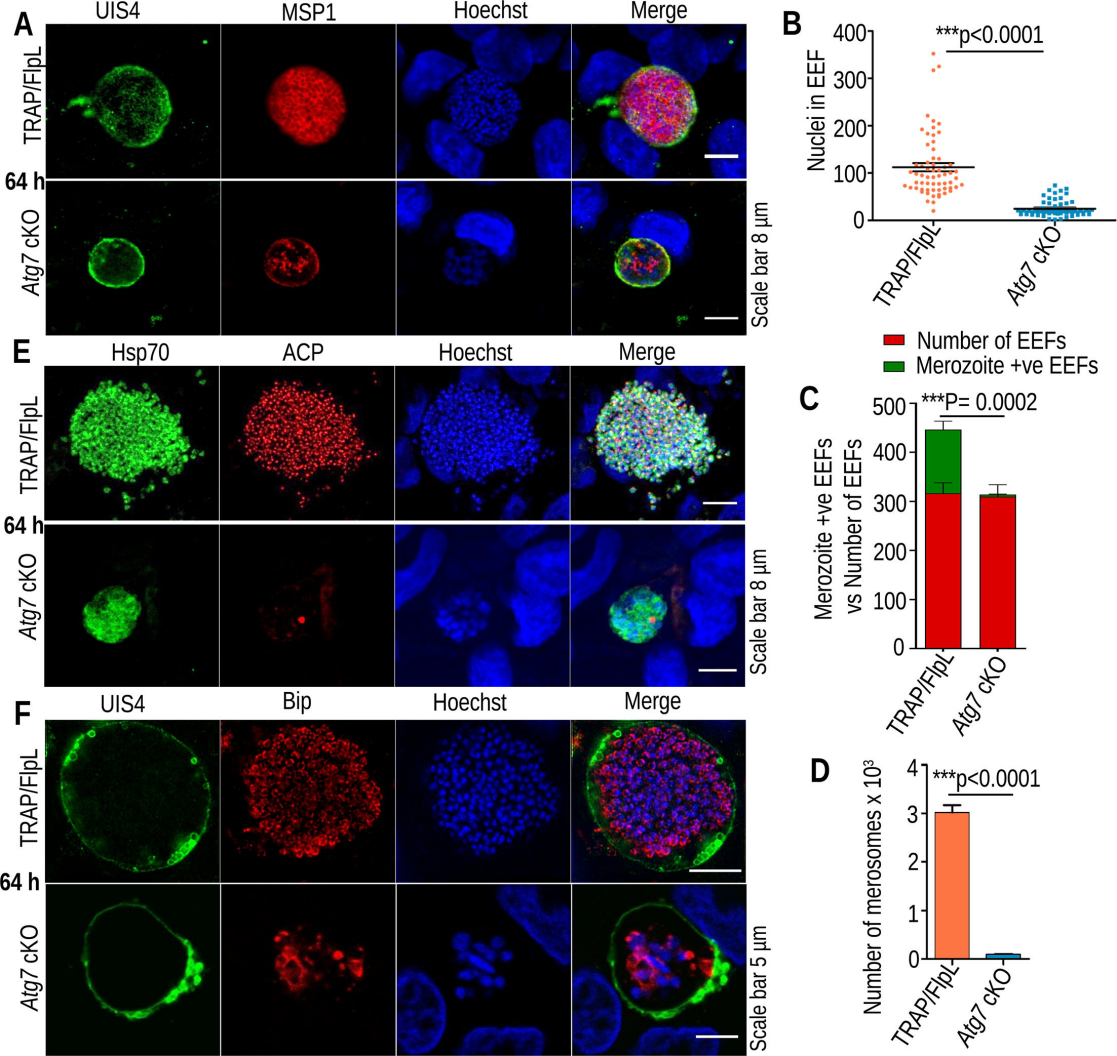
*Atg7* cKO parasites show impaired organelle biogenesis and fail to mature into hepatic merozoites. (**A**) *Atg7* cKO or TRAP/FlpL EEFs fixed at 64 hpi were stained with MSP1 and UIS4 antibodies. TRAP/FlpL mature EEFs showed clear nuclear segregation, with each nucleus surrounded by MSP1, whereas the *Atg7* cKO parasites failed to segregate their nuclei, and MSP1 development was impaired. (**B**) Nuclei count in *Atg7* cKO or TRAP/FlpL EEFs. Hoechst-stained nuclei images were acquired under a confocal microscope, and nuclei were counted using an ImageJ cell counting tool. Data were pooled from three independent experiments (*n* = 70) (significant difference, *P* < 0.0001, Student’s *t*-test). The nuclei were stained with Hoechst 33342. (**C**) The EEFs harvested at 64 hpi were observed under a fluorescence microscope for MSP1 staining. Merozoites were rarely detected in the *Atg7* cKO parasites. A significant difference was observed between TRAP/FlpL and *Atg7* cKO parasites (***P = 0.0002, Student’s *t*-test). (**D**) The number of detached cells (merosomes) in *Atg7* cKO or TRAP/FlpL-infected HepG2 cultures. Data are presented as the mean ± SEM. *n* = 3 biological replicates (significant difference, *P* < 0.0001, Student’s *t*-test). (**E and F**) EEFs fixed at 64 hpi were immunostained with ACP/Hsp70 and Bip/UIS4 antibodies. EEFs showed impaired organelle biogenesis, and both apicoplast and ER development were aborted in *Atg7* cKO parasites.

Arrested growth of the *Atg7* cKO EEFs was further confirmed by observing merozoite development by staining with MSP1 antibody, which was found to be impaired, including the nuclei count (Fig. 4A and B). We rarely found the merozoite-positive EEFs in *Atg7* cKO parasites compared to TRAP/FlpL (Fig. 4C). After the maturation of parasites in the liver, they are released in the form of merosomes (3). We enumerated the merosome numbers in TRAP/FlpL; however, due to the absence of merosomes in the *Atg7* cKO parasites, the collected supernatant was divided equally and injected into five Swiss mice (Fig. 4D). The TRAP/FlpL group injected with 10 merosomes became patent on day 5; however, all the mice injected with *Atg7* cKO culture supernatant remained negative during the observation period (Table 2). These findings indicated that in the absence of Atg7, parasites failed to develop into infective merozoites, which explains their inability to initiate blood-stage infection. Next, to assess whether loss of Atg7 function had a role in organelle biogenesis during liver-stage development, apicoplasts and endoplasmic reticulum (ER) were visualized using IFA. We found extensive branching of the apicoplast and ER in TRAP/FlpL parasites, while this branching was lost in the *Atg7* cKO parasites, suggesting impaired development of organelles in the mutant parasites (Fig. 4E and F). These findings implicate the loss of the conjugation of Atg8 to the apicoplast/endomembrane in the absence of Atg7. Atg8 localization on endomembranes confers many unique roles in their mammalian counterparts, such as membrane tethering, hemifusion, and expansion of autophagosome membranes (16). Both autophagosomes and apicoplasts originate in the endomembrane system, and the disruption of Atg8 in *P. falciparum/T. gondii* results in a defect in apicoplast biogenesis (14, 19–21, 29).

**TABLE 2:** Merosome formation was impaired in *Atg7 c*KO parasites^*a*^

Experiment	Parasites	No. of merosome injected	Mice pos/per injected	Pre-patent period (days)
1	TRAP Flp/L	10	5/5	5
	*Atg7* cKO SC2	—	0/5	N/A
2	TRAP Flp/L	10	5/5	5
	*Atg7* cKO SC2	—	0/5	N/A

^*a*^ Swiss mice were injected intravenously with merosomes or culture supernatant.

### Atg7-mediated conjugation of Atg8 is required for biogenesis of the apicoplast

**Fig 5:**
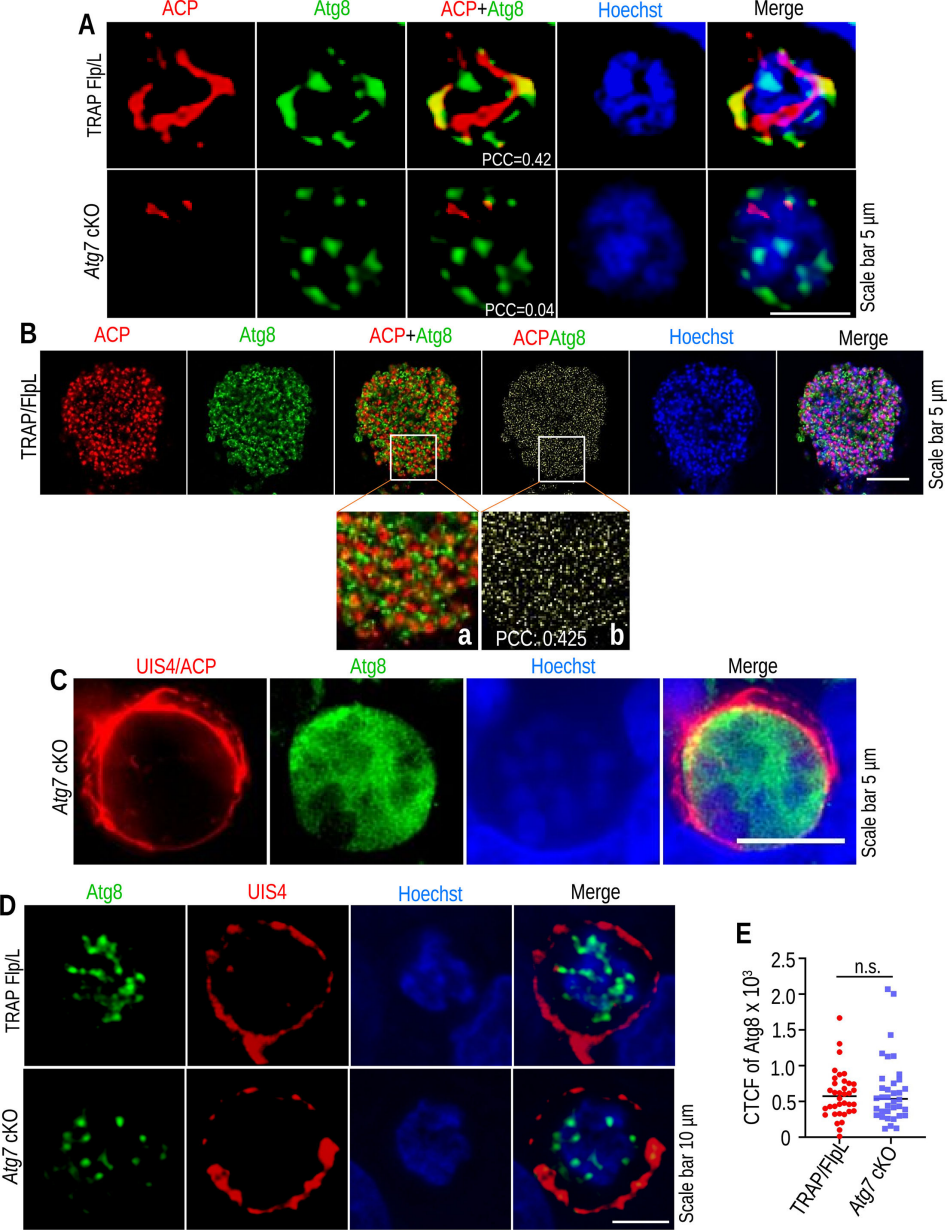
Atg8 localization on the membrane is required for apicoplast branching. (**A**) TRAP/FlpL and *Atg7* cKO EEFs harvested at 20 hpi were immunostained with anti-ACP and anti-Atg8 antibodies. *Atg7* cKO parasites failed to conjugate Atg8 to the apicoplast membrane. (**B**) TRAP/FlpL merosomes harvested at 64 hpi were immunostained with anti-ACP and anti-Atg8 antibodies. A significant portion of Atg8 was found to be localized on the apicoplast (PCC: 0.425). Inset images (**a, b**) show a zoomed portion of conjugated Atg8 on the apicoplast. The nuclei were stained with Hoechst 33342. (**C**) *Atg7* cKO EEFs harvested at 64 hpi were immunostained with anti-ACP and anti-Atg8 antibodies. EEFs were identified using an anti-UIS4 antibody. We did not have other options, so we used rabbit-raised anti-UIS4 and anti-ACP antibodies in this IFA. Anti-UIS4 antibody stains parasitophorous vacuolar membrane (PVM), which is present mainly on the periphery and does not interfere much with the apicoplast signal, evident from the other UIS4-stained EEFs. We observed apicoplast formation without Atg7-mediated Atg8 lipidation, but it did not undergo extensive branching and subsequent expansion. The nuclei were stained with Hoechst 33342. (**D**) TRAP/FlpL and *Atg7* cKO EEFs harvested at 24 hpi were immunostained with anti-UIS4 and anti-Atg8 antibodies. (**E**) Quantifying Atg8 fluorescence signals in TRAP/FlpL or *Atg7* cKO EEFs fixed at 24 hpi. Corrected total cell fluorescence (CTCF) of Atg8 in TRAP/FlpL and *Atg7* cKO parasites were comparable (no significant difference, *P* = 0.619, Student’s *t*-test).

With significant growth defects observed at the late stages of exoerythrocytic schizogony, we next analyzed the Atg8 distribution pattern in the liver stages of parasitic development. *Atg7* cKO and TRAP/FlpL EEFs fixed at 20 and 64 hpi were immunostained with anti-ACP and anti-Atg8 antibodies to visualize Atg8 lipidation on the branched apicoplast structures. We found Atg8-mediated apicoplast expansion and division in TRAP/FlpL parasites, and this conjugation and branching were impaired in *Atg7* cKO parasites (Fig. 5A through C). A previous study reported that overexpression of Atg8 by *P. berghei* liver forms has detrimental effects on EEF development (30). Next, we harvested EEFs at 24 hpi and immunostained them with anti-Atg8 antibodies to quantify the Atg8 level (Fig. 5D). We found a similar level of Atg8 in TRAP/FlpL and *Atg7* cKO parasites (Fig. 5E). These results indicate that due to the lack of Atg8 conjugation, the apicoplast failed to expand and segregate during liver-stage development. The *Atg7* cKO phenotype is due to the lack of Atg8 lipidation, not the overexpression of Atg8.

### Atg7 is essential for the exocytosis of unnecessary superfluous organelles during liver-stage development

**Fig 6:**
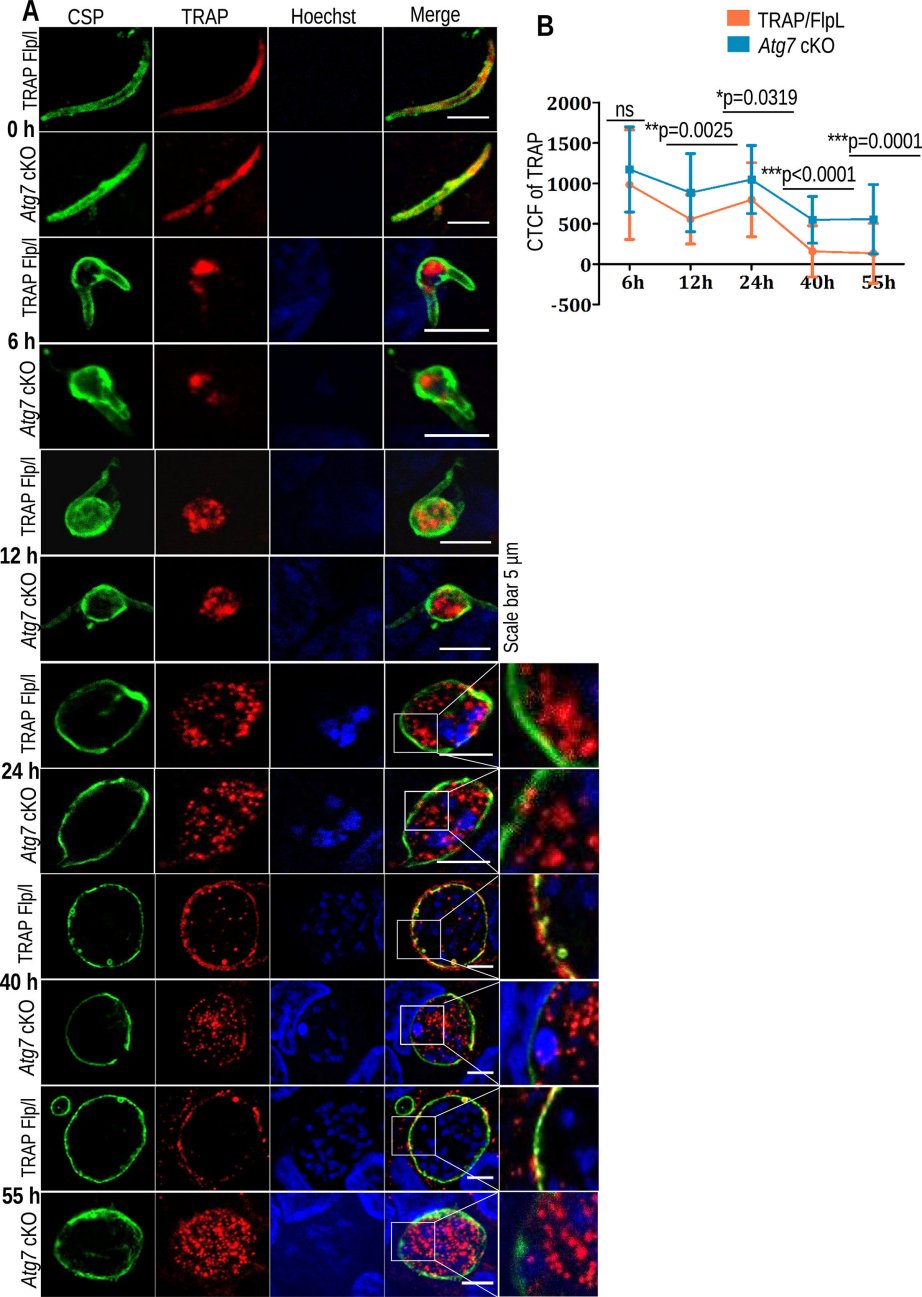
*Atg7* cKO parasites failed to exocytose micronemes during liver-stage development. (**A**) HepG2 cultures infected with *Atg7* cKO or TRAP/FlpL sporozoites were fixed at different time points. The EEFs were stained with TRAP antibody to track the movement of micronemes and were stained with CSP antibody to identify the EEFs. The images show the aggregation of the micronemes by 6 to 12 hpi, and by 24 h, the micronemes began to divert toward the PV membrane of the parasites in TRAP/FlpL EEFs, while in *Atg7* cKO parasites, the micronemes remained centered. By 40 to 55 hpi, most of the micronemal content was excreted out of the parasite cytosol, while a significant number of *Atg7* cKO EEFs retained their micronemes. (**B**) Quantification of TRAP fluorescence signals in TRAP/FlpL or *Atg7* cKO EEFs was fixed at 6–55 hpi. The EEFs were stained with anti-TRAP antibody, and its signal inside the PV membrane was calculated as corrected total cell fluorescence (CTCF) (*n* = 30–35 EEFs). Data are presented as the mean ± SD. *n* = 3 biological replicates (no significant difference at 6 h [*P* = 0.2325]; significant difference at 12 h [*P* = 0.0025], 24 h [*P* = 0.0319], 40 h [*P* < 0.0001], and 55 h [*P* = 0.0001]; Student’s *t*-test). The nuclei were stained with Hoechst 33342.

During parasite development in the liver-stage, a series of morphological changes occur that involve the mechanical elimination of the secretory organelles, which are no longer required post-hepatocyte invasion by the parasite. Previous studies have reported the presence of membrane whorls enclosing the IMC, rhoptries, and micronemal contents secreted out in the extracellular matrix, which are further degraded by the proteolytic enzymes released by the parasite (28). Here, we monitored the elimination pattern of micronemes by using thrombospondin-related anonymous protein (TRAP) antibodies during the liver-stage development of *Atg7* cKO and TRAP/FlpL parasites. We first started with its identification in sporozoites and then followed its track in EEFs. We found the same distribution pattern of micronemes in TRAP/FlpL and *Atg7* cKO parasites at 0 h (Fig. 6A). By 6 to 12 hpi in TRAP/FlpL parasites, micronemal content was found to accumulate in the center of EEFs as the parasite achieved spherical morphology (Fig. 6A). *Atg7* cKO parasites also showed a similar distribution pattern. Still, there was a significant increase in the intensity of TRAP signals at 12 hpi as calculated by corrected total cell fluorescence (CTCF) (Fig. 6B). TRAP/FlpL EEFs observed at 24 hpi showed TRAP-enriched organelles migrating toward the PV membrane. At the same time, they remained in the center in the *Atg7* cKO parasites. (Fig. 6A). TRAP/FlpL EEFs fixed at 40 and 55 hpi showed 70% to 83% elimination of micronemal content, while it was approximately 36% and 40% in *Atg7* cKO parasites. Quantification of TRAP signals revealed an increase in CTCF values post-12 h, indicating a severe defect in the exocytosis of these rudimentary organelles in *Atg7* cKO parasites (Fig. 6B).

## DISCUSSION

In this study, we show that *Pb*Atg7, which is an intermediate enzyme of the Atg8 conjugation pathway, plays an essential role in parasite blood and liver stages. *Atg7* cKO parasites failed to lipidate Atg8 on the membrane and showed impaired liver-stage development due to a defect in microneme clearance and failed apicoplast branching. Our finding is consistent with previous studies showing the essentiality of the Atg8 conjugation pathway in *P. falciparum* asexual blood stages (21, 24, 31). Many autophagy studies on apicomplexans have shown the discrete localization of Atg8 on the apicoplast, and loss of Atg8 directly affects its biogenesis (14, 19, 20, 23, 25, 29).

Apicoplast localization of Atg8 in apicomplexan parasites has diversified its role apart from autophagy. We have demonstrated the direct involvement of Atg7-mediated Atg8 lipidation on the branching of apicoplasts during liver-stage development. In the absence of Atg7-mediated Atg8 lipidation, apicoplast can still form but does not undergo extensive branching and subsequent expansion, due to which liver-stage parasites fail to mature into hepatic merozoites. This implies that Atg8 lipidation on endomembranes might be a source of membrane supply for the extensive branching of apicoplasts during the replicative phase of parasite development. A study reported accelerated development of the apicoplast network due to overexpression of Atg8 during liver-stage development. The unusually fast expansion of apicoplast from the beginning of liver infection led to a prematurely large network in early schizonts that starts dismantling in mature schizonts (30). Another study on *T. gondii* showed that apicoplast genome segregation is synchronized with nuclear division and that the apicoplast interacts with centrosomes during division (32). This interaction might be mediated via Atg8, as its downregulation led to improper segregation of apicoplasts into individual tachyzoites (23). Although *Plasmodium* parasites lack centrioles, they have a centrosome-like structure, and Atg8 might interact with it, which enables the positioning of the apicoplast during division (33, 34).

The existence of apicoplasts has been proven to be crucial for parasite survival. The organelle has lost its photosynthetic abilities but has retained several important metabolic pathways, such as the biosynthesis of fatty acids, isoprenoids, heme, and iron-sulfur clusters (35). Loss of the integrity of the apicoplast or interference with any of its biosynthetic pathways would affect the supply of such important metabolites that affect parasite development. In a study, the loss of apicoplast protein PALM resulted in parasite attenuation in the liver-stage that further failed to develop into merozoites and did not initiate blood-stage infection (36). Similar detrimental effects on growth were observed when genes of the FASII pathway were deleted, leading to parasite arrest in liver-stage forms (37–39). Impaired apicoplast expansion hampers the subcellular distribution and supply of lipids that might indirectly affect ER differentiation, as suggested by their closed apposition and vesicle sharing (40).

Furthermore, our study implicates the role of Atg7 in maintaining cellular homeostasis via a special secretory autophagy pathway present in the *Plasmodium* parasite. In this pathway, organelle-enclosed endomembranous vesicles are exocytosed by a secretory pathway and later degraded by an extracellular enzymatic process (41–43). We found that TRAP-filled micronemal contents tend to move toward the PV membrane and be exocytosed by 40–55 hpi in wild-type parasites; however, this process was impaired in *Atg7* cKO parasites, suggesting the role of the Atg8 conjugation pathway in the elimination of secretory organelles during liver-stage development (1, 30). Mammalian homologs of Atg8, LC3, and GABARAP have been shown to interact with microtubules through their LIR motifs, which might enable the movement of autophagosomes and GABA receptor-like vesicles (44–47). Similarly, Atg8 on micronemal vesicles might interact with microtubules and enable the movement and elimination of the vesicle out of the parasite lumen.

Overall, our study showed the importance of the Atg7 in *P. berghei* blood and liver-stage development. The pathway enables the clearance of micronemal vesicles from the parasite cytosol, has also been diversified, and plays a role in the biogenesis of organelles during exoerythrocytic schizogony. Finally, the essentiality of *Plasmodium* Atg7 during blood and liver stages holds the potential to develop a multistage drug to alleviate malaria.

## MATERIALS AND METHODS

### Parasites and cell lines

*Plasmodium berghei* ANKA (MRA 311) was obtained from BEI resources, USA. The marker-free transgenic *P. berghei* ANKA line expressing FlpL under *Plasmodium* stage-specific promotor-TRAP was used for the generation of conditional knockout parasites (48). *Plasmodium* sporozoites were obtained by infecting female *Anopheles stephensi* mosquitoes as described previously (48). Human liver hepatocellular carcinoma (HepG2) was regularly maintained in Dulbecco's modified Eagle's medium (DMEM; Sigma) supplemented with 10% fetal bovine serum (FBS; Sigma), 0.2% NaHCO_3_ (Sigma), 1% sodium pyruvate (Genetix), and 1% penicillin‒streptomycin (Invitrogen) at 37°C with 5% CO_2_. We routinely tested cell lines for mycoplasma contamination.

### Mice

Female Swiss albino mice (6–8 wk old) were used for routine parasite infections and passages, and C57BL/6 mice 6–8 wk old were used for sporozoite *in vivo* infection.

### Generation of transgenic and knockout parasites

For the generation of *Pb*Atg7-3×HA-mCherry transgenic parasites, two fragments, F1 (0.66 kb) and F2 (0.62 kb), were amplified using primers 1632/1633 and 1634/1324 and were sequentially cloned into the pBC-3×HA-mCherry-hDHFR plasmid at *Xho*I*/Bgl*II and *Not*I*/Asc*I (NEB), respectively. The plasmid was linearized with *Xho*I*/Asc*I and transfected into *P. berghei* ANKA purified schizonts as described previously (49). In an attempt to generate direct knockout of *Pb*Atg7, two fragments, F3 (0.54 kb) and F4 (0.62 kb), were amplified using primers 1321/1322 and 1323/1324 and were sequentially cloned into pBC-GFP-yFCU-hDHFR at blunt-ended *Sal*I and *Not*I/*Asc*I sites, respectively. The plasmid was linearized with *Xho*I/*Asc*I restriction digestion and transfected into *P. berghei* ANKA schizonts (49). For the generation of the conditional knockout of *Pb*Atg7 (PBANKA_0922200), three fragments, F5 (1.03 kb), F6 (0.16 kb), and F7 (0.62 kb), were amplified using primers 1325/1326, 1327/1328, and 1353/1330, respectively. All three fragments, F5, F6, and F7, were sequentially cloned into a p3’TRAP-flirte-hDHFR plasmid at *Hind*III*/Not*I, EcoRV, and *Sal*I*/Kpn*I restriction sites, respectively. For swapping the 3′UTR of the gene with TRAP, 12 bp was added to the F6 fragment to continue the 3′UTR function. The cloned plasmid was linearized by restriction digestion with *Hind*III*/Kpn*I and transfected into *P. berghei* ANKA TRAP/FlpL purified schizonts (48, 50). All transfected parasites were selected by oral administration of pyrimethamine (0.07 mg/mL, Sigma) for six consecutive days, and resistant parasites were collected and genotyped. For 5′ and 3′ integrations, the primers 1399/1216 and 1215/1400 were used for *Atg7* cKO, and primers 2022/1218 and 1215/1400 for Atg7-3×HA-mCherry transgenic parasites, respectively (for primers, see Table S1 at https://doi.org/10.6084/m9.figshare.27641523.v2). Clonal lines of the cKO and transgenics were obtained by limiting dilutions of the parasites in Swiss mice and used for further analysis.

### Analysis of asexual blood-stage propagation

To analyze the effect of swapping the 3’UTR of the gene with the TRAP 3′UTR during blood-stage development, an equal number of iRBCs of Atg7 cKO and TRAP/FlpL parasites were i.v. injected into Swiss mice. The growth was monitored daily by making Giemsa-stained blood smears.

### Analysis of parasite development in the mosquito

For parasite transmission in the mosquito, female *Anopheles stephensi* mosquitoes were fed on Swiss mice infected either with *Atg7* cKO or TRAP/FlpL parasites and were kept in an environmental chamber maintained at 19°C with 80% relative humidity. On day 14 post-blood meal, the mosquito midgut was dissected and imaged under a Nikon Eclipse 80i microscope using a 10× (numerical aperture [NA], 0.25; air) objective with no filter, and the sporulation pattern and oocyst numbers were determined. To determine the oocyst sporozoite number, mosquito midguts were crushed and spun at 50 × *g* for 4 min, the supernatant was collected, and the sporozoite number was counted using a hemocytometer under a Nikon phase contrast microscope using a 40× (NA 0.75; air) objective with no filter. To achieve the optimal excision efficiency of the flirted *Atg7* locus by the flippase enzyme, mosquito cages were transferred to 25°C on day 17 post-blood meal. Salivary glands were dissected on days 21–23, and the sporozoite number per mosquito was enumerated. To check the excised flirted locus, genomic DNA was isolated from sporozoites using a genomic DNA purification kit (Promega) and was genotyped using primers 1409/1400 (for primers, see Table S1 at https://doi.org/10.6084/m9.figshare.27641523.v2) (48).

### Sporozoite *in vivo* infectivity

To assess the *in vivo* infectivity of sporozoites, C57BL/6 mice were i.v. injected with 5,000 sporozoites (five mice per group). Daily examination of Giemsa-stained blood smears estimated the onset of blood-stage infection. The patent mice were genotyped using primers 1409/1400. To estimate the liver-stage parasite biomass, another group of C57BL/6 mice was i.v. inoculated with 5,000 *Atg7* cKO or TRAP/FlpL sporozoites, and the liver was harvested and homogenized at different time points in TRIzol reagent (Himedia). Total RNA was isolated using the manufacturer’s instructions.

### cDNA synthesis and real-time PCR

Randomly primed cDNA was synthesized using 1 µg of RNA in a reverse transcriptase reaction containing 1× PCR buffer, 0.5 mM dNTPs, 5 mM MgCl2, 20 U RNase inhibitor, 2.5 µM random hexamers, and 50 U reverse transcriptase (Applied Biosystems) in a thermocycler (Eppendorf). The resulting cDNA was used for the quantification of *Pb*18s rRNA, *Pb*MSP1, and mouse GAPDH transcripts as described previously (51) using SYBR green reagent (Takara) with primer pairs 1195/1196, 1219/1220, and 1193/1194, respectively. The transcript number of parasites was expressed as the normalized value by taking the ratio of *Pb*18s rRNA/mGAPDH or *Pb*MSP1/mGAPDH. All data were obtained using a CFX Opus 96 real-time PCR system (Bio-Rad) (for primers, see Table S1 at https://doi.org/10.6084/m9.figshare.27641523.v2).

### Sporozoite *in vitro* infectivity

The hepatocyte infectivity and development assay was performed as previously described (52). Briefly, HepG2 cells were plated on 48-well or 24-well plate cultures at a density of 55,000 or 100,000 cells per well, respectively. For the invasion assay, salivary gland sporozoites (10,000 sporozoites per well) were added to a 48-well culture plate and allowed to invade for 2 h. The cells were fixed with 4% paraformaldehyde (PFA) for 20 min at room temperature (RT). For the development assay, salivary gland sporozoites (5,000 sporozoites per well) were added to the 48-well plate culture and maintained as described previously (53). The culture was fixed at 24, 40, 55, and 64 hpi using 4% PFA for 20 min at RT. For the merosome assay, salivary gland sporozoites (30,000 sporozoites per well) were added to the 24-well plate culture, and at 64 hpi, the culture was observed live. The supernatant was collected and quantified using a hemocytometer. Swiss mice were intravenously injected (10 merosomes per mouse) to check merosome infectivity. Giemsa-stained blood smears were observed to indicate the onset of blood-stage infection. The genotyping of infected mice was performed using primers 1409/1400 (for primers, see Table S1 at https://doi.org/10.6084/m9.figshare.27641523.v2). To estimate the parasite biomass, HepG2 cultures infected in a 24-well plate were harvested in TRIzol reagent at different time points, RNA was isolated and reverse transcribed, and transcripts were quantified using real-time PCR as described above.

### Immunofluorescence assay

Fixed cultures were washed twice in phosphate-buffered saline (PBS) and blocked with 1% bovine serum albumin (BSA)/PBS for the invasion assay. The extracellular and intracellular sporozoites were immunostained with anti-circumsporozoite protein (CSP) mouse antibody (54) (diluted 1 µg/mL) before and after permeabilization as described previously (52). Primary antibodies were then detected using anti-mouse IgG conjugated to Alexa Fluor 594 or Alexa Fluor 488 (diluted 1:500; Invitrogen), and nuclei were stained with Hoechst 33342 (Invitrogen), mounted using prolong diamond antifade reagent (Life Technologies), and visualized under a Nikon Eclipse 80i fluorescence microscope (100× [NA 1.30; oil] objective). For EEF development, fixed cultures were washed twice in PBS, permeabilized with 0.1% Triton X-100 (Sigma) for 10 min at RT, washed again in PBS, and blocked with 1% BSA/PBS. Primary antibodies were diluted in 1% BSA/PBS and incubated for 1 h at RT, and then the wells were washed three times with PBS. Primary antibody signals were then detected using anti-mouse, anti-rat, or anti-rabbit Alexa Fluor-conjugated IgG as described above. To analyze the EEF number and area, EEFs were stained with Upregulated in infectious sporozoites gene 4 (UIS4) (55) (diluted 1:1,000, rabbit polyclonal) and heat shock protein 70 (Hsp70) (56) (diluted 1:500, mouse monoclonal). The EEFs were counted using a Nikon Eclipse 80i fluorescence microscope, and the area of the EEFs was measured using NIS-D software. Merozoite formation was visualized using merozoite surface protein 1 (MSP1) antibody (57) (diluted 1:500, mouse monoclonal). Apicoplast and ER development was visualized using acyl carrier protein (ACP) antibody (diluted 1:1,000, rabbit polyclonal [58]) and Bip (diluted 1:500, rat polyclonal, BEI Resources), respectively. Anti-Atg8 antibody developed in the rat (diluted 1:200) (22) was used to analyze the Atg8 expression and distribution pattern. Anti-Atg8 signals were revealed using Alexa Fluor 488-conjugated anti-rat IgG (diluted 1:500; Invitrogen). A TRAP antibody was used to visualize micronemes. Affinity-purified polyclonal rabbit antibody against *P. berghei* TRAP was developed by GenScript Inc., Piscataway, NJ, USA, against the peptide sequence CAEPAKPAEPAEPAE. For microneme distribution, EEFs were fixed at different time points and immunolabeled with TRAP (diluted 1:200, rabbit polyclonal) and CSP (54) (diluted 1:1,000, mouse monoclonal) antibodies. The TRAP and CSP signals were revealed using Alexa Fluor 594-conjugated anti-rabbit IgG and Alexa Fluor 488-conjugated anti-mouse IgG, respectively (diluted 1:500; Invitrogen). Nuclei were stained with Hoechst 33342, and the coverslips were mounted using Prolong Diamond antifade reagent (Life Technologies). Representative images were acquired using FV1000 software on a confocal laser scanning microscope (Olympus BX61WI) using a UPlanSAPO 100× (NA 1.4, oil) or 63× (NA 0.25, oil) objective. Leica DM 3000 LED microscope 100× (NA 1.25, oil) objective was used for calculating CTCF (59) of EEFs using ImageJ 1.52 (60, 61), as given in the equation *CTCF = IntDen* – *(A × MBF*). IntDen is the integrated fluorescence density of the selected cell, *A* is the area of the selected cell, and MBF is the mean background fluorescence of the image. All measurements were plotted using GraphPad Prism version 8.0. The calculated CTCF values were used to derive the percent microneme exocytosis.

### Immunolocalization

For localization studies, different parasite stages were fixed with 4% PFA (Sigma) for 20 min at RT. The parasites were permeabilized with chilled methanol at 4°C for 15 min, blocked with 1% BSA/PBS, and immunolabeled with anti-mCherry (diluted 1:500, Novus Biologicals) and anti-Hsp70 (diluted 1:500) antibodies for 2 h at RT. The mCherry and Hsp70 signals were revealed using Alexa Fluor 594-conjugated anti-rabbit IgG and Alexa Fluor 488-conjugated anti-mouse IgG, respectively (diluted 1:500; Invitrogen). Nuclei were stained with Hoechst 33342. The images were taken using a Leica 3000 LED microscope and pseudocolored using ImageJ fiji “Lookup Table” features. The colocalization of the protein was estimated using the Pearson correlation coefficient calculated using the ImageJ “JACop” plugin.

### Antibody generation

The *P. falciparum* Atg8 (PF3D7_1019900)-expressing clone was used to express the recombinant protein (62). The pET32a-*Pf*Atg8 plasmid, which expresses Atg8 as a thioredoxin-His fusion (Trx-His-Atg8), was transformed into *Escherichia coli* BL21 (DE3) cells, and expression was induced using isopropyl b-D-thiogalactopyranoside (SRL) and further purified as previously described (62). The rat was primed with purified protein in complete Freund’s adjuvant and boosted twice with incomplete Freund’s adjuvant. The rats were bled, and the serum was collected for further analysis. Anti-PfAtg8 antibodies generated in rats are cross-reactive with the PbAtg8 protein as previously described (22).

### Nuclei count

The quantification of nuclei per EEF was performed using ImageJ Fiji software. TRAP/FlpL and *Atg7* cKO 64 hpi EEFs stained with UIS4 and Hoechst 33342 were used for quantification. Hsp70 staining was used to visualize the parasite, and the DNA centers were counted using the “multipoint” feature. The images were deconvoluted for segmentation and then analyzed using ImageJ’s thresholding and watershed segmentation features.

### Statistical analysis

Data are presented as the means ± standard error of the means (SEMs) or mean ± standard deviation (SD). Statistical analysis was performed using GraphPad Prism version 8.0. Statistical significance between the two groups was analyzed using an unpaired two-tailed *t*-test or one-way analysis of variance (ANOVA). A *P* value less than 0.05 was considered statistically significant.

## Data Availability

All data are available within this article, and raw data are available from the corresponding author upon reasonable request. Materials generated in this study are available from the corresponding author on request.
